# Methyl 3-*O*-α-d-mannopyranosyl β-d-glucopyran­oside tetra­hydrate

**DOI:** 10.1107/S1600536808021454

**Published:** 2008-07-31

**Authors:** Lars Eriksson, Göran Widmalm

**Affiliations:** a Division of Structural Chemistry, Arrhenius Laboratory, Stockholm University, SE-106 91 Stockholm, Sweden; b Department of Organic Chemistry, Arrhenius Laboratory, Stockholm University, SE-106 91 Stockholm, Sweden

## Abstract

The title compound, C_13_H_24_O_11_·4H_2_O, forms extended hydrogen-bonded networks. These are present between disaccharides, but not as inter-residue hydrogen bonds, as well as to water mol­ecules that in addition form an inter­molecular chain of hydrogen bonds. The conformation of the disaccharide is described by the glycosidic torsion angles ϕ_H_ = −34° and ψ_H_ = −5°. Macroscopically, the disaccharide was observed to be hygroscopic.

## Related literature

For related literature, see: Cremer & Pople (1975 ▶); Eriksson & Widmalm (2005 ▶); Eriksson *et al.* (1997 ▶, 2000 ▶, 2002 ▶); Färnbäck *et al.* (2003 ▶, 2008 ▶); Hassel & Ottar (1947 ▶); Huskens (2006 ▶); Jansson *et al.* (1990 ▶); Juaristi & Cuevas (1992 ▶); Odelius *et al.* (1995 ▶); Vishnyakov *et al.* (2000 ▶).
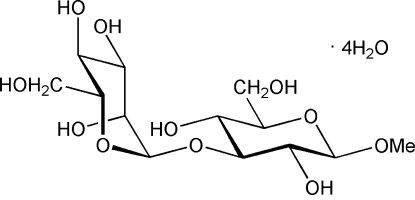


## Experimental

### 

#### Crystal data

C_13_H_24_O_11_·4H_2_O
*M* *_r_* = 428.39
Monoclinic, 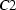

*a* = 18.275 (3) Å
*b* = 7.7293 (12) Å
*c* = 13.910 (3) Å
β = 97.87 (2)°
*V* = 1946.4 (6) Å^3^
*Z* = 4
Mo *K*α radiation
μ = 0.14 mm^−1^
*T* = 291 (2) K
0.40 × 0.30 × 0.15 mm

#### Data collection

Stoe IPDS diffractometer
Absorption correction: numerical (*X-RED*; Stoe & Cie, 1997 ▶) *T*_min_ = 0.95, *T*_max_ = 0.98
8973 measured reflections
2017 independent reflections
1706 reflections with *I* > 2σ(*I*)
*R*_int_ = 0.037

#### Refinement

*R*[*F* ^2^ > 2σ(*F* ^2^)] = 0.025
*wR*(*F* ^2^) = 0.059
*S* = 0.99
2017 reflections
287 parameters
9 restraints
H atoms treated by a mixture of independent and constrained refinement
Δρ_max_ = 0.13 e Å^−3^
Δρ_min_ = −0.14 e Å^−3^

### 

Data collection: *EXPOSE* (Stoe & Cie, 1997 ▶); cell refinement: *CELL* (Stoe & Cie, 1997 ▶); data reduction: *INTEGRATE* (Stoe & Cie, 1997 ▶); program(s) used to solve structure: *SHELXS97* (Sheldrick, 2008 ▶); program(s) used to refine structure: *SHELXL97* (Sheldrick, 2008 ▶); molecular graphics: *DIAMOND* (Bergerhoff, 1996 ▶); software used to prepare material for publication: *PLATON* (Spek, 2003 ▶).

## Supplementary Material

Crystal structure: contains datablocks I, global. DOI: 10.1107/S1600536808021454/om2251sup1.cif

Structure factors: contains datablocks I. DOI: 10.1107/S1600536808021454/om2251Isup2.hkl

Additional supplementary materials:  crystallographic information; 3D view; checkCIF report

## Figures and Tables

**Table 1:** Selected torsion angles (°)

O5*m*—C5*m*—C6*m*—O6*m*	−64.9 (2)
C4*m*—C5*m*—C6*m*—O6*m*	57.2 (2)
O5*g*—C1*g*—O1*g*—C7	−71.2 (2)
C2*g*—C1*g*—O1*g*—C7	168.7 (2)
O5*m*—C1*m*—O3*g*—C3*g*	85.18 (19)
C2*m*—C1*m*—O3*g*—C3*g*	−151.40 (15)
C4*g*—C3*g*—O3*g*—C1*m*	112.63 (18)
C2*g*—C3*g*—O3*g*—C1*m*	−124.11 (18)
O5*g*—C5*g*—C6*g*—O6*g*	−69.7 (2)
C4*g*—C5*g*—C6*g*—O6*g*	50.1 (2)
H1*m*—C1*m*—O3*g*—C3*g*	−34
C1*m*—O3*g*—C3*g*—H3*g*	−5

**Table 2:** Hydrogen-bond geometry (Å, °)

*D*—H⋯*A*	*D*—H	H⋯*A*	*D*⋯*A*	*D*—H⋯*A*
O2*m*—H2*m*1⋯O3*m*^i^	0.82	1.96	2.732 (2)	156
O3*m*—H3*m*1⋯O6*m*^ii^	0.82	1.89	2.705 (2)	172
O4*m*—H4*m*1⋯O*W*4^iii^	0.82	2.03	2.803 (2)	158
O6*m*—H6*m*⋯O*W*3^iv^	0.82	2.00	2.796 (2)	166
O2*g*—H2*g*1⋯O4*m*^v^	0.82	2.25	2.848 (2)	130
O2*g*—H2*g*1⋯O3*m*^v^	0.82	2.43	3.140 (2)	145
O4*g*—H4*g*1⋯O*W*2^vi^	0.82	1.91	2.733 (2)	177
O6*g*—H6*g*⋯O*W*1^vi^	0.82	2.00	2.794 (2)	162
O*W*1—H11⋯O4*g*	0.94 (2)	1.80 (2)	2.736 (2)	174 (4)
O*W*1—H12⋯O*W*2	0.97 (2)	1.92 (3)	2.834 (2)	156 (2)
O*W*2—H21⋯O*W*3	0.92 (2)	1.98 (2)	2.866 (2)	161 (4)
O*W*2—H22⋯O2*g*^vi^	0.90 (3)	2.06 (3)	2.915 (2)	159 (4)
O*W*3—H31⋯O1*g*^vii^	0.91 (3)	1.94 (3)	2.814 (2)	163 (4)
O*W*3—H32⋯O*W*4	0.90 (2)	1.92 (2)	2.807 (2)	167 (4)
O*W*4—H41⋯O6*g*^vii^	0.89 (2)	2.04 (2)	2.916 (2)	168 (3)
O*W*4—H42⋯O2*m*^vi^	0.89 (2)	1.88 (3)	2.747 (2)	163 (4)

Symmetry codes: (i) 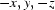; (ii) 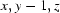; (iii) 
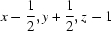
; (iv) 
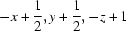
; (v) 
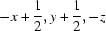
; (vi) 
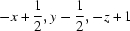
; (vii) 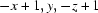.

**Fig. 1.**
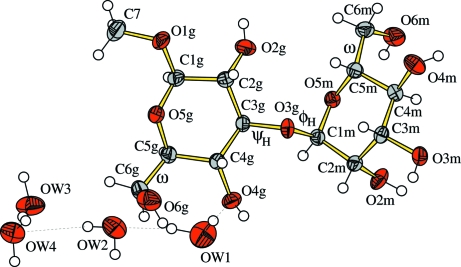
Molecular structure of the title compound, showing 50% probability displacement ellipsoids and the atom numbering scheme. H atoms are shown as small spheres of arbitrary radii.

**Fig. 2.**
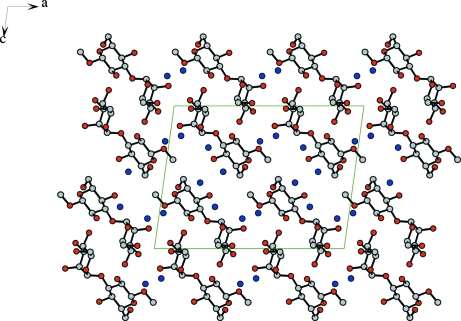
Crystal packing of the title compound, showing slightly more than one unit cell, viewed along the b axis direction. The water molecules between the sugar residues are situated in channels along the b-direction.

## supplementary crystallographic information

### Comment

Carbohydrates in biological systems, in the case of N-linked glycans of glycoproteins the result of post-translational modifications, are of functional significance due to *e.g.* their influence on protein stability. Furthermore, highly specific epitopes are formed by oligosaccharides present as glycoconjugates. The information contents in carbohydrate structures are indeed very large as a consequence of the immense numbers of permutations possible by combining different linkages and anomeric configurations of the sugar residues. It is of particular importance that the often weak carbohydrate interactions function by resorting to multivalent interactions upon cell-cell recognition (Huskens, 2006).

The major degrees of freedom in an oligosaccharide are described by the torsion angles φ_H_, ψ_H_, and ω. For the title compound the two former are present at the glycosidic α-(1 → 3)-linkage with φ_H_ being defined by H1m—C1m—O3g—C3g and ψ_H_ by C1m—O3g—C3g—H3g. The ω torsion angle, defined by O5—C5—C6—O6, refers to the conformation of the hydroxymethyl group of each sugar residue. The structure is described as the *exo*-anomeric conformation with φ_H_ = -34°, which, as a result of stereoelectronic effects, is characteristic of sugars in a cyclic form (Fig. 1). For the title compound the presence of the *endo*-anomeric effect (Juaristi & Cuevas, 1992) is evident from the difference in C—O bond lengths at the anomeric positions of the α-D-Man*p* residue having the axial bond C1m—O3g = 1.409 (2) Å and the β-D-Glc*p* residue having the equatorial bond C1g—O1g = 1.402 (2) Å, *i.e.*, the bond with the axial electronegative atom is longer than the corresponding equatorial one, in complete agreement with *ab initio* data of model compounds (Odelius *et al.*, 1995). At the glycosidic linkage ψ_H_ = -5°, leading to an almost eclipsed conformation and as a result the inter-residue distance across the glycosidic linkage for the proton pair H1m—H3g becomes short, only 2.12 Å.

The conformations of the hydroxymethyl groups are described by one of the three rotamers, *gauche*-*trans*, *gauche*-*gauche*, or *trans*-*gauche* with respect to the orientation of C6—O6 to C5—O5 and to C5—C4, respectively. In the present case both the mannopyranosyl and the glucopyranosyl residues show the *gg* conformation for their hydroxymethyl groups with ω = -64.9 (2)° and ω = -69.7 (2)°, respectively. This conformation is one of the two anticipated rotamers for the monosaccharides in the title compound, since both have an equatorial hydroxyl group at C4, which precludes the *tg* rotamer as a result of a non-favorable 1,3-diaxial interaction known as the Hassel-Ottar effect (Hassel & Ottar, 1947).

The calculated Cremer & Pople (1975) parameters show that both the mannose and glucose rings are close to the expected chair conformation, *i.e.*^4^C_1_. The parameters for the mannose ring are [Q=0.555 (2) Å, θ=3.0 (2) ° and φ=302 (3) °] and for the glucose ring [Q=0.575 (2) Å, θ=10.0 (2) ° and φ=327 (1) °].

The title compound was quite hygroscopic. This fact is consistent with the relatively high water content in the crystal of the title disaccharide. In our previous structural studies on disaccharide crystals the number of water molecules ranged from zero to three per disaccharide (Eriksson *et al.* 1997, 2000, 2002, 2005; Färnbäck *et al.* 2003, 2008). All hydroxyl groups and all H atoms of the four water molecules are hydrogen bond donors and the structure is stabilized by an elaborate hydrogen bond network. The four water molecules can be considered as lying in channels along the b-direction between the sugar residues as shown in Fig. 2. Previous conformational studies on the title compound that focused on solution patterns in binary aqueous solvent mixtures indicated that an inter-residue hydrogen bond was present between O6m as the donor atom and O2g as the acceptor atom (Vishnyakov *et al.* 2000). This was possible when the ω torsion angle of the mannosyl residue had the gt conformation. However, in the present crystal structure the *exo*-cyclic hydroxymethyl groups of the glucosyl residue as well as that in the mannosyl residue have the gg conformation, the latter of which precludes the intra-molecular hydrogen bond. Further analysis of the hydrogen bonding patterns showed that O6m acts as a donor to OW3. The O6g atom, on the other hand, acts as a donor to OW1, which acts as a donor to OW2, continued in a donor-acceptor relationship to OW3, and in an analogous way to OW4. Finally, the latter water molecule acts as a donor to the acceptor O6g in another molecule. Thus, the water-mediated chain starts from one glucosyl residue and ends at a symmetry related glucosyl residue. Along the 'chain of water molecules' various atoms of the sugar residues act as hydrogen bond donors and acceptors. The close proximity of O2g in one molecule and O3m and O4m in a symmetry related molecule at distances of 3.140 (2) Å and 2.848 (2) Å, respectively, indicate that a bifurcated hydrogen bond is present with O2g as the donor atom. The triangle formed by the three oxygen atoms is almost isosceles with an O3m^v^—O2g—O4m^v^ [symmetry code (v): -*x* + 1/2,*y* + 1/2,-*z*] angle of 56.09 (4)°.

### Experimental

The synthesis of the title compound was described by Jansson *et al.* (1990). The disaccharide was crystallized by slow evaporation from a mixture of water, ethanol and acetonitrile (1:1:1) at ambient temperature. The absolute configuration of each sugar residue is known from the starting compounds used in the synthesis.

### Refinement

The hydrogen atoms were geometrically placed and constrained to ride on the parent atom. The C—H bond distances are 0.96 Å for CH_3_, 0.97 Å for CH_2_, 0.98 Å for CH. The *U*_iso_(H) = 1.5 *U*_eq_(C) for the CH_3_ and 1.2 *U*_eq_(C) for all other H atoms. Due to the abscence of significant anomalous scatterers, the value of the Flack parameter (Flack, 1983) was not meaningful, thus the 1707 Friedel equivalents were included in the merging process (MERG 3 in *SHELXL97*). The H atoms of the water molecule were located from difference density map and the d(O—H) were restrained to retain the previously known geometry of the water molecule. The hydrogen atoms of the water molecule were given *U*_iso_(H) = 1.5*U*_eq_(O).

### Crystal data

**Table:**

C_13_H_24_O_11_·4H_2_O	*F*_000_ = 920
*M**_r_* = 428.39	*D*_x_ = 1.462 Mg m^−^^3^
Monoclinic, *C*2	Mo *K*α radiation λ = 0.71073 Å
Hall symbol: C 2y	Cell parameters from 1641 reflections
*a* = 18.275 (3) Å	θ = 2.3–25.9º
*b* = 7.7293 (12) Å	µ = 0.14 mm^−^^1^
*c* = 13.910 (3) Å	*T* = 291 (2) K
β = 97.87 (2)º	Block, colourless
*V* = 1946.4 (6) Å^3^	0.40 × 0.30 × 0.15 mm
*Z* = 4	

### Data collection

**Table:**

Stoe IPDS diffractometer	2017 independent reflections
Radiation source: fine-focus sealed tube	1706 reflections with *I* > 2σ(*I*)
Monochromator: graphite	*R*_int_ = 0.037
Detector resolution: 6 pixels mm^-1^	θ_max_ = 25.9º
*T* = 291(2) K	θ_min_ = 2.3º
φ scans	*h* = −22→22
Absorption correction: numerical(X-RED; Stoe & Cie, 1997)	*k* = −9→9
*T*_min_ = 0.95, *T*_max_ = 0.98	*l* = −16→17
8973 measured reflections	

### Refinement

**Table:**

Refinement on *F*^2^	Secondary atom site location: difference Fourier map
Least-squares matrix: full	Hydrogen site location: difference Fourier map
*R*[*F*^2^ > 2σ(*F*^2^)] = 0.025	H atoms treated by a mixture of independent and constrained refinement
*wR*(*F*^2^) = 0.059	*w* = 1/[σ^2^(*F*_o_^2^) + (0.0391*P*)^2^] where *P* = (*F*_o_^2^ + 2*F*_c_^2^)/3
*S* = 0.99	(Δ/σ)_max_ < 0.001
2017 reflections	Δρ_max_ = 0.13 e Å^−^^3^
287 parameters	Δρ_min_ = −0.14 e Å^−^^3^
9 restraints	Extinction correction: SHELXL97 (Sheldrick, 2008), Fc^*^=kFc[1+0.001xFc^2^λ^3^/sin(2θ)]^-1/4^
Primary atom site location: structure-invariant direct methods	Extinction coefficient: 0.0054 (9)

### Special details

**Table:**

Geometry. All e.s.d.'s (except the e.s.d. in the dihedral angle between two l.s. planes) are estimated using the full covariance matrix. The cell e.s.d.'s are taken into account individually in the estimation of e.s.d.'s in distances, angles and torsion angles; correlations between e.s.d.'s in cell parameters are only used when they are defined by crystal symmetry. An approximate (isotropic) treatment of cell e.s.d.'s is used for estimating e.s.d.'s involving l.s. planes.
Refinement. Refinement of *F*^2^ against ALL reflections. The weighted *R*-factor *wR* and goodness of fit *S* are based on *F*^2^, conventional *R*-factors *R* are based on *F*, with *F* set to zero for negative *F*^2^. The threshold expression of *F*^2^ > σ(*F*^2^) is used only for calculating *R*-factors(gt) *etc*. and is not relevant to the choice of reflections for refinement. *R*-factors based on *F*^2^ are statistically about twice as large as those based on *F*, and *R*- factors based on ALL data will be even larger.

### Fractional atomic coordinates and isotropic or equivalent isotropic displacement parameters (Å^2^)

**Table:**

	*x*	*y*	*z*	*U*_iso_*/*U*_eq_	
C1m	0.16629 (10)	0.4371 (3)	0.18727 (14)	0.0237 (4)	
H1m	0.1530	0.4374	0.2531	0.028*	
C2m	0.11852 (10)	0.3031 (3)	0.12731 (15)	0.0253 (4)	
H2m	0.1312	0.1869	0.1525	0.030*	
O2m	0.04476 (7)	0.3436 (2)	0.14055 (11)	0.0354 (4)	
H2m1	0.0161	0.2829	0.1047	0.053*	
C3m	0.13077 (10)	0.3138 (3)	0.02186 (15)	0.0231 (4)	
H3m	0.1812	0.2752	0.0169	0.028*	
O3m	0.08036 (8)	0.2061 (2)	−0.03918 (11)	0.0297 (3)	
H3m1	0.0874	0.1046	−0.0234	0.044*	
C4m	0.12196 (11)	0.4985 (3)	−0.01623 (14)	0.0246 (4)	
H4m	0.0703	0.5351	−0.0193	0.030*	
O4m	0.14398 (9)	0.5051 (2)	−0.11052 (11)	0.0390 (4)	
H4m1	0.1121	0.5547	−0.1478	0.059*	
C5m	0.17184 (10)	0.6189 (3)	0.05093 (14)	0.0251 (4)	
H5m	0.2233	0.5838	0.0501	0.030*	
O5m	0.15526 (7)	0.60426 (18)	0.14856 (9)	0.0244 (3)	
C6m	0.16458 (11)	0.8075 (3)	0.02356 (17)	0.0312 (5)	
H6m1	0.1970	0.8747	0.0705	0.037*	
H6m2	0.1809	0.8234	−0.0394	0.037*	
O6m	0.09124 (8)	0.8723 (2)	0.01954 (13)	0.0393 (4)	
H6m	0.0769	0.8585	0.0724	0.059*	
C1g	0.41344 (10)	0.5571 (3)	0.33556 (15)	0.0276 (5)	
H1g	0.3968	0.6384	0.3821	0.033*	
O1g	0.47702 (7)	0.6210 (2)	0.30165 (10)	0.0340 (4)	
C2g	0.35400 (10)	0.5359 (3)	0.24839 (15)	0.0288 (5)	
H2g	0.3743	0.4734	0.1967	0.035*	
O2g	0.32707 (8)	0.6996 (2)	0.21340 (13)	0.0441 (4)	
H2g1	0.3564	0.7431	0.1808	0.066*	
C3g	0.28842 (10)	0.4364 (3)	0.27749 (14)	0.0244 (4)	
H3g	0.2609	0.5128	0.3159	0.029*	
O3g	0.24034 (6)	0.38309 (19)	0.19220 (10)	0.0268 (3)	
C4g	0.31150 (10)	0.2760 (3)	0.33673 (14)	0.0251 (4)	
H4g	0.3316	0.1905	0.2954	0.030*	
O4g	0.24809 (7)	0.2055 (2)	0.37211 (11)	0.0330 (4)	
H4g1	0.2383	0.1108	0.3470	0.050*	
C5g	0.37025 (10)	0.3225 (3)	0.42143 (15)	0.0278 (4)	
H5g	0.3501	0.4101	0.4615	0.033*	
O5g	0.43192 (7)	0.3957 (2)	0.38142 (11)	0.0310 (3)	
C6g	0.39763 (12)	0.1712 (3)	0.48482 (17)	0.0400 (6)	
H6g1	0.4398	0.2075	0.5304	0.048*	
H6g2	0.3590	0.1348	0.5218	0.048*	
O6g	0.41839 (9)	0.0293 (2)	0.42994 (15)	0.0498 (5)	
H6g	0.3866	−0.0460	0.4273	0.075*	
C7	0.53302 (12)	0.6785 (4)	0.37756 (18)	0.0435 (6)	
H71	0.5146	0.7745	0.4109	0.065*	
H72	0.5760	0.7139	0.3499	0.065*	
H73	0.5459	0.5857	0.4225	0.065*	
OW1	0.19300 (11)	0.2972 (3)	0.53816 (17)	0.0612 (5)	
H11	0.2119 (18)	0.274 (5)	0.480 (2)	0.092*	
H12	0.2364 (15)	0.320 (5)	0.585 (2)	0.092*	
OW2	0.28912 (11)	0.3894 (3)	0.70793 (15)	0.0564 (5)	
H21	0.3355 (13)	0.361 (5)	0.737 (3)	0.085*	
H22	0.2615 (18)	0.332 (5)	0.747 (3)	0.085*	
OW3	0.44168 (10)	0.3766 (3)	0.78999 (16)	0.0577 (5)	
H31	0.4706 (19)	0.463 (4)	0.773 (3)	0.086*	
H32	0.4659 (19)	0.275 (4)	0.787 (3)	0.086*	
OW4	0.52138 (11)	0.0800 (3)	0.75157 (14)	0.0540 (5)	
H41	0.5331 (19)	0.061 (5)	0.6924 (19)	0.081*	
H42	0.4990 (18)	−0.009 (4)	0.776 (3)	0.081*	

### Atomic displacement parameters (Å^2^)

**Table:**

	*U*^11^	*U*^22^	*U*^33^	*U*^12^	*U*^13^	*U*^23^
C1m	0.0207 (8)	0.0279 (11)	0.0223 (10)	0.0007 (8)	0.0020 (7)	0.0011 (8)
C2m	0.0215 (9)	0.0253 (11)	0.0285 (11)	−0.0011 (8)	0.0014 (8)	0.0026 (9)
O2m	0.0218 (6)	0.0514 (10)	0.0336 (8)	−0.0102 (7)	0.0067 (6)	−0.0122 (7)
C3m	0.0188 (8)	0.0236 (10)	0.0264 (10)	0.0030 (8)	0.0015 (7)	−0.0035 (9)
O3m	0.0329 (7)	0.0208 (7)	0.0322 (8)	0.0033 (6)	−0.0064 (6)	−0.0024 (6)
C4m	0.0258 (9)	0.0262 (11)	0.0218 (10)	0.0034 (8)	0.0026 (8)	0.0008 (9)
O4m	0.0518 (9)	0.0410 (10)	0.0261 (8)	0.0069 (8)	0.0118 (7)	0.0037 (7)
C5m	0.0227 (9)	0.0258 (10)	0.0273 (10)	0.0015 (8)	0.0049 (8)	0.0029 (9)
O5m	0.0255 (7)	0.0238 (7)	0.0236 (7)	0.0009 (6)	0.0023 (5)	−0.0015 (6)
C6m	0.0305 (10)	0.0253 (11)	0.0377 (12)	−0.0014 (9)	0.0045 (9)	0.0040 (10)
O6m	0.0382 (8)	0.0271 (8)	0.0528 (10)	0.0086 (7)	0.0070 (7)	0.0084 (8)
C1g	0.0230 (9)	0.0328 (12)	0.0277 (10)	−0.0026 (8)	0.0059 (8)	0.0006 (9)
O1g	0.0244 (7)	0.0456 (9)	0.0323 (8)	−0.0101 (7)	0.0056 (6)	−0.0001 (7)
C2g	0.0243 (9)	0.0347 (12)	0.0276 (11)	−0.0026 (9)	0.0046 (8)	0.0048 (9)
O2g	0.0347 (8)	0.0448 (10)	0.0522 (11)	−0.0040 (8)	0.0033 (7)	0.0247 (9)
C3g	0.0220 (9)	0.0288 (11)	0.0214 (10)	−0.0010 (8)	−0.0002 (7)	−0.0002 (9)
O3g	0.0202 (6)	0.0349 (8)	0.0238 (7)	0.0044 (6)	−0.0027 (5)	−0.0025 (7)
C4g	0.0218 (9)	0.0272 (11)	0.0263 (10)	−0.0003 (8)	0.0029 (7)	0.0021 (9)
O4g	0.0304 (7)	0.0347 (9)	0.0351 (9)	−0.0087 (7)	0.0082 (6)	−0.0006 (8)
C5g	0.0239 (9)	0.0328 (11)	0.0263 (10)	−0.0007 (8)	0.0023 (8)	0.0020 (9)
O5g	0.0213 (6)	0.0361 (8)	0.0348 (8)	−0.0011 (6)	0.0006 (6)	0.0062 (7)
C6g	0.0327 (11)	0.0499 (16)	0.0357 (13)	0.0002 (10)	−0.0017 (9)	0.0120 (11)
O6g	0.0454 (9)	0.0424 (10)	0.0628 (12)	0.0112 (8)	0.0112 (9)	0.0138 (9)
C7	0.0316 (11)	0.0531 (16)	0.0444 (14)	−0.0129 (11)	0.0001 (9)	−0.0019 (12)
OW1	0.0568 (11)	0.0722 (14)	0.0581 (13)	−0.0008 (10)	0.0205 (9)	−0.0107 (12)
OW2	0.0614 (11)	0.0459 (11)	0.0601 (12)	0.0062 (10)	0.0014 (9)	0.0157 (11)
OW3	0.0507 (10)	0.0498 (12)	0.0775 (14)	−0.0013 (9)	0.0270 (10)	0.0152 (11)
OW4	0.0581 (11)	0.0581 (13)	0.0474 (11)	−0.0042 (9)	0.0128 (9)	0.0088 (10)

### Geometric parameters (Å, °)

**Table:**

C1m—O5m	1.403 (2)	C2g—C3g	1.525 (3)
C1m—O3g	1.409 (2)	C2g—H2g	0.9800
C1m—C2m	1.527 (3)	O2g—H2g1	0.8200
C1m—H1m	0.9800	C3g—O3g	1.437 (2)
C2m—O2m	1.420 (2)	C3g—C4g	1.516 (3)
C2m—C3m	1.516 (3)	C3g—H3g	0.9800
C2m—H2m	0.9800	C4g—O4g	1.428 (2)
O2m—H2m1	0.8200	C4g—C5g	1.524 (3)
C3m—O3m	1.431 (2)	C4g—H4g	0.9800
C3m—C4m	1.523 (3)	O4g—H4g1	0.8200
C3m—H3m	0.9800	C5g—O5g	1.439 (2)
O3m—H3m1	0.8200	C5g—C6g	1.508 (3)
C4m—O4m	1.425 (2)	C5g—H5g	0.9800
C4m—C5m	1.529 (3)	C6g—O6g	1.417 (3)
C4m—H4m	0.9800	C6g—H6g1	0.9700
O4m—H4m1	0.8200	C6g—H6g2	0.9700
C5m—O5m	1.436 (2)	O6g—H6g	0.8200
C5m—C6m	1.508 (3)	C7—H71	0.9600
C5m—H5m	0.9800	C7—H72	0.9600
C6m—O6m	1.425 (3)	C7—H73	0.9600
C6m—H6m1	0.9700	OW1—H11	0.94 (2)
C6m—H6m2	0.9700	OW1—H12	0.97 (2)
O6m—H6m	0.8200	OW2—H21	0.92 (2)
C1g—O1g	1.402 (2)	OW2—H22	0.90 (2)
C1g—O5g	1.421 (3)	OW3—H31	0.91 (2)
C1g—C2g	1.522 (3)	OW3—H32	0.90 (2)
C1g—H1g	0.9800	OW4—H41	0.89 (2)
O1g—C7	1.437 (3)	OW4—H42	0.89 (2)
C2g—O2g	1.419 (3)	H1m—H3g	2.12
			
O5m—C1m—O3g	112.22 (16)	C1g—O1g—C7	113.67 (16)
O5m—C1m—C2m	111.93 (15)	O2g—C2g—C1g	110.69 (18)
O3g—C1m—C2m	107.40 (16)	O2g—C2g—C3g	107.01 (16)
O5m—C1m—H1m	108.4	C1g—C2g—C3g	110.13 (17)
O3g—C1m—H1m	108.4	O2g—C2g—H2g	109.7
C2m—C1m—H1m	108.4	C1g—C2g—H2g	109.7
O2m—C2m—C3m	112.45 (16)	C3g—C2g—H2g	109.7
O2m—C2m—C1m	105.16 (16)	C2g—O2g—H2g1	109.5
C3m—C2m—C1m	110.02 (15)	O3g—C3g—C4g	107.92 (16)
O2m—C2m—H2m	109.7	O3g—C3g—C2g	109.85 (16)
C3m—C2m—H2m	109.7	C4g—C3g—C2g	112.73 (16)
C1m—C2m—H2m	109.7	O3g—C3g—H3g	108.8
C2m—O2m—H2m1	109.5	C4g—C3g—H3g	108.8
O3m—C3m—C2m	111.96 (16)	C2g—C3g—H3g	108.8
O3m—C3m—C4m	108.06 (15)	C1m—O3g—C3g	115.46 (15)
C2m—C3m—C4m	111.41 (16)	O4g—C4g—C3g	108.71 (15)
O3m—C3m—H3m	108.4	O4g—C4g—C5g	110.03 (16)
C2m—C3m—H3m	108.4	C3g—C4g—C5g	109.96 (16)
C4m—C3m—H3m	108.4	O4g—C4g—H4g	109.4
C3m—O3m—H3m1	109.5	C3g—C4g—H4g	109.4
O4m—C4m—C3m	108.92 (16)	C5g—C4g—H4g	109.4
O4m—C4m—C5m	108.71 (16)	C4g—O4g—H4g1	109.5
C3m—C4m—C5m	109.40 (15)	O5g—C5g—C6g	108.43 (16)
O4m—C4m—H4m	109.9	O5g—C5g—C4g	107.44 (16)
C3m—C4m—H4m	109.9	C6g—C5g—C4g	114.28 (18)
C5m—C4m—H4m	109.9	O5g—C5g—H5g	108.9
C4m—O4m—H4m1	109.5	C6g—C5g—H5g	108.9
O5m—C5m—C6m	107.01 (16)	C4g—C5g—H5g	108.9
O5m—C5m—C4m	110.16 (15)	C1g—O5g—C5g	111.60 (14)
C6m—C5m—C4m	114.19 (17)	O6g—C6g—C5g	112.14 (19)
O5m—C5m—H5m	108.4	O6g—C6g—H6g1	109.2
C6m—C5m—H5m	108.4	C5g—C6g—H6g1	109.2
C4m—C5m—H5m	108.4	O6g—C6g—H6g2	109.2
C1m—O5m—C5m	113.43 (15)	C5g—C6g—H6g2	109.2
O6m—C6m—C5m	113.55 (17)	H6g1—C6g—H6g2	107.9
O6m—C6m—H6m1	108.9	C6g—O6g—H6g	109.5
C5m—C6m—H6m1	108.9	O1g—C7—H71	109.5
O6m—C6m—H6m2	108.9	O1g—C7—H72	109.5
C5m—C6m—H6m2	108.9	H71—C7—H72	109.5
H6m1—C6m—H6m2	107.7	O1g—C7—H73	109.5
C6m—O6m—H6m	109.5	H71—C7—H73	109.5
O1g—C1g—O5g	107.66 (15)	H72—C7—H73	109.5
O1g—C1g—C2g	107.69 (16)	H11—OW1—H12	105 (3)
O5g—C1g—C2g	111.29 (17)	H21—OW2—H22	100 (3)
O1g—C1g—H1g	110.0	H31—OW3—H32	109 (3)
O5g—C1g—H1g	110.0	H41—OW4—H42	114 (4)
C2g—C1g—H1g	110.0		
			
O5m—C1m—C2m—O2m	−67.68 (19)	O1g—C1g—C2g—C3g	169.66 (17)
O3g—C1m—C2m—O2m	168.72 (15)	O5g—C1g—C2g—C3g	51.9 (2)
O5m—C1m—C2m—C3m	53.6 (2)	O2g—C2g—C3g—O3g	72.5 (2)
O3g—C1m—C2m—C3m	−70.0 (2)	C1g—C2g—C3g—O3g	−167.17 (16)
O2m—C2m—C3m—O3m	−55.7 (2)	O2g—C2g—C3g—C4g	−167.15 (17)
C1m—C2m—C3m—O3m	−172.58 (15)	C1g—C2g—C3g—C4g	−46.8 (2)
O2m—C2m—C3m—C4m	65.4 (2)	O5m—C1m—O3g—C3g	85.18 (19)
C1m—C2m—C3m—C4m	−51.4 (2)	C2m—C1m—O3g—C3g	−151.40 (15)
O3m—C3m—C4m—O4m	−64.6 (2)	C4g—C3g—O3g—C1m	112.63 (18)
C2m—C3m—C4m—O4m	172.00 (15)	C2g—C3g—O3g—C1m	−124.11 (18)
O3m—C3m—C4m—C5m	176.70 (14)	O3g—C3g—C4g—O4g	−66.67 (19)
C2m—C3m—C4m—C5m	53.31 (19)	C2g—C3g—C4g—O4g	171.85 (17)
O4m—C4m—C5m—O5m	−175.11 (16)	O3g—C3g—C4g—C5g	172.82 (14)
C3m—C4m—C5m—O5m	−56.29 (19)	C2g—C3g—C4g—C5g	51.3 (2)
O4m—C4m—C5m—C6m	64.5 (2)	O4g—C4g—C5g—O5g	−178.89 (16)
C3m—C4m—C5m—C6m	−176.72 (17)	C3g—C4g—C5g—O5g	−59.2 (2)
O3g—C1m—O5m—C5m	61.40 (19)	O4g—C4g—C5g—C6g	60.8 (2)
C2m—C1m—O5m—C5m	−59.44 (19)	C3g—C4g—C5g—C6g	−179.52 (18)
C6m—C5m—O5m—C1m	−174.57 (16)	O1g—C1g—O5g—C5g	178.17 (15)
C4m—C5m—O5m—C1m	60.76 (18)	C2g—C1g—O5g—C5g	−64.0 (2)
O5m—C5m—C6m—O6m	−64.9 (2)	C6g—C5g—O5g—C1g	−169.21 (17)
C4m—C5m—C6m—O6m	57.2 (2)	C4g—C5g—O5g—C1g	66.8 (2)
O5g—C1g—O1g—C7	−71.2 (2)	O5g—C5g—C6g—O6g	−69.7 (2)
C2g—C1g—O1g—C7	168.7 (2)	C4g—C5g—C6g—O6g	50.1 (2)
O1g—C1g—C2g—O2g	−72.2 (2)	H1m—C1m—O3g—C3g	−34
O5g—C1g—C2g—O2g	170.02 (16)	C1m—O3g—C3g—H3g	−5

### Hydrogen-bond geometry (Å, °)

**Table:**

*D*—H···*A*	*D*—H	H···*A*	*D*···*A*	*D*—H···*A*
O2m—H2m1···O3m^i^	0.82	1.96	2.732 (2)	156
O3m—H3m1···O6m^ii^	0.82	1.89	2.705 (2)	172
O4m—H4m1···OW4^iii^	0.82	2.03	2.803 (2)	158
O6m—H6m···OW3^iv^	0.82	2.00	2.796 (2)	166
O2g—H2g1···O4m^v^	0.82	2.25	2.848 (2)	130
O2g—H2g1···O3m^v^	0.82	2.43	3.140 (2)	145
O4g—H4g1···OW2^vi^	0.82	1.91	2.733 (2)	177
O6g—H6g···OW1^vi^	0.82	2.00	2.794 (2)	162
OW1—H11···O4g	0.94 (2)	1.80 (2)	2.736 (2)	174 (4)
OW1—H12···OW2	0.97 (2)	1.92 (3)	2.834 (2)	156 (2)
OW2—H21···OW3	0.92 (2)	1.98 (2)	2.866 (2)	161 (4)
OW2—H22···O2g^vi^	0.90 (3)	2.06 (3)	2.915 (2)	159 (4)
OW3—H31···O1g^vii^	0.91 (3)	1.94 (3)	2.814 (2)	163 (4)
OW3—H32···OW4	0.90 (2)	1.92 (2)	2.807 (2)	167 (4)
OW4—H41···O6g^vii^	0.89 (2)	2.04 (2)	2.916 (2)	168 (3)
OW4—H42···O2m^vi^	0.89 (2)	1.88 (3)	2.747 (2)	163 (4)

Symmetry codes: (i) −*x*, *y*, −*z*; (ii) *x*, *y*−1, *z*; (iii) *x*−1/2, *y*+1/2, *z*−1; (iv) −*x*+1/2, *y*+1/2, −*z*+1; (v) −*x*+1/2, *y*+1/2, −*z*; (vi) −*x*+1/2, *y*−1/2, −*z*+1; (vii) −*x*+1, *y*, −*z*+1.
